# Effect of the Boron Concentration in Irrigation Water on the Elemental Composition of Edible Parts of Tomato, Green Bean, Potato, and Cabbage Grown on Soils With Different Textures

**DOI:** 10.3389/fpls.2021.658892

**Published:** 2021-06-14

**Authors:** Márk Rékási, Péter Ragályi, Anna Füzy, Nikolett Uzinger, Péter Dobosy, Gyula Záray, Nóra Szűcs-Vásárhelyi, András Makó, Tünde Takács

**Affiliations:** ^1^Institute for Soil Sciences, Centre for Agricultural Research, Budapest, Hungary; ^2^Institute of Aquatic Ecology, Centre for Ecological Research, Budapest, Hungary

**Keywords:** irrigation, element uptake, translocation, vegetables, nutritional value

## Abstract

The most important environmental source of boron (B) contamination is irrigation water. The data on the effect of B on the elemental composition in the edible parts of vegetables are scarce. A greenhouse pot experiment investigated the effect of irrigation water containing 0.1 and 0.5 mg/L B on the biomass, elemental (e.g., B, Mg, K, Fe, Cu, and Zn) composition, and photosynthetic parameters of tomato (*Solanum lycopersicum*), green bean (*Phaseolus vulgaris*), potato (*Solanum tuberosum*), and cabbage (*Brassica oleracea*) plants grown on 10 kg of sand, silty sand, or silty soil. The biomass of the edible part was unaffected by B treatment. The soil type determined the effect of B irrigation on the elemental composition of vegetables. The B content increased by 19% in tomatoes grown on silty soil. The 0.1 mg/L B treatment facilitated tomato fruit ripening on all soils, and the 0.5 mg/L B treatment doubled its chlorophyll content index (CCI) on silty soil. The 0.5 mg/L B treatment negatively affected the nutritional value of green beans on all soils, decreasing the Fe and K contents by an average of 83 and 34%, respectively. The elemental composition of potato was unaffected by the treatments, but the CCI of potato leaves increased in the 0.5 mg/L B treatment by 26%. The B content was increased by 39% in cabbages grown on light-textured soils. In conclusion, B concentration of up to 0.5 mg/L in irrigation water had no significant beneficial or adverse effect on the investigated vegetables, but 0.1 mg/L B treatment could shorten tomato fruit maturation time on B-poor soils. The B levels in vegetables remained suitable for human consumption.

## Introduction

The three quality criteria for irrigation water are decisive for the potential threat to crop production, namely, salinity, sodicity, and toxicity. Regarding the third criterion, the main characteristic water components that may cause adverse physiological effects in plants are chloride and boron (B; Rhoades, 1972). On the one hand, the application of B-rich irrigation water (B irrigation) is a global issue and the most important B pollution source in the environment (Türker et al., 2019). On the other hand, a study by FAO revealed that B deficiency is a worldwide problem, affecting about 8 million ha (Tariq and Mott, 2007).

Boron (B) is both a potential hazard and an essential micronutrient for vascular plants. It is not classified as an essential nutrient in the human diet (Naghii et al., 1996; Rainey et al., 1999), but B deficiency affects the skeleton and brain physiology and modifies the metabolism of several nutrients (Nielsen, 1996). The daily intake ranges from 0.02 to >9 mg/day; the consumption of B above 10 mg/day may be toxic (Rainey et al., 1999; EFSA, 2018), but an intake of at least 1 mg B/day is recommended (Nielsen, 1997). The major B sources are legumes, fresh vegetables, and fruits (Naghii et al., 1996).

In plants, B deficiency causes biochemical, physiological, and anatomical aberrations. B plays a role in carbohydrate transport, cell wall and membrane synthesis and function, nucleic acid and hormone metabolism, and the growth of the apical meristem (Howe, 1998; Bolaños et al., 2004; Pereira et al., 2021). Of all the nutrients, B has the narrowest optimal concentration in a range between toxicity and deficiency (Rees et al., 2011). The tolerance of plants to B is highly dependent on soil properties and irrigation management, which influence the availability of B (Bingham, 1973). Vascular plants absorb B mostly by passive diffusion, especially in the case of adequate or high availability of B. Under these circumstances, the absorption of B is mostly determined by the B concentration in the soil solution and the transpiration rate. However, if the availability of B is low, the transportation of active membrane will also occur in the plants (Dannel et al., 2002).

For plants, the main source of B is the soil, the B content of which depends mostly on the elemental composition of the parent material. The total B concentration in soils ranges from 1 to 467 mg/kg, with an average value of 9–85 mg/kg. B may be adsorbed on either organic or inorganic surfaces in the soil, but these bonds are weak (Kabata-Pendias and Pendias, 2001). In addition, B in mineral forms is not readily available for plants (Nable et al., 1997). In soils with higher CaCO_3_ content and pH value, B is less available for plants, probably due mainly to the pH that facilitates the formation of the tetrahedral anionic form of boric acid (Goldberg, 1997; Parks and Edwards, 2005). Soils with high organic matter (OM) content and a pH of less than 7.3 may contain more available B than that is required for plant nutrition (Berger and Truog, 1939). The soil texture, moisture content, and temperature also affect the availability of B. The higher soil clay content results in a higher adsorption capacity of B, which increases with increasing pH and reaches its maximum at pH 8 (Goldberg, 1997). Thus, the addition of B with irrigation water may influence the elemental composition and growth of plants in different ways on different soils.

The amount of B available in the soil determines the plant uptake of B, which in turn influences the elemental composition of the plant and thus the nutritional value of edible plant parts. The data on the B tolerance of various crops are available in the literature, but information on the effect of B irrigation on elemental composition, and hence, the nutritional value of edible plant parts is scarce. The other shortcoming of the available literature is that most experiments were performed under hydroponic conditions or on artificial growth media, thus preventing investigations on the effect of soil properties that may modify the availability of B. Parks et al. (1944) found that, on quartz sand, the elemental composition of tomato leaves changed as a function of B supplies. The sign of changes in element contents of plants was dependent on the soil B concentration. Eggert and von Wirén (2016) demonstrated that B fertilization may improve the microelement uptake of oilseed rape seedlings in growth media that are poor in B. Tariq and Mott (2006) reported the increases in the concentration of B, Zn, and Cu and the decreases in Fe, Mn, and Mo in radish plants that are grown on acid-washed sand as the B concentration of the nutrient solution rose. The leaves of tobacco grown on peat growth medium showed an increase in Mn and Fe concentration and a decrease in Cu and Zn concentration as a function of the B concentration in the irrigation water (López-Lefebre et al., 2002). The effect of B irrigation on the elemental concentration of maize grown on quartz sand was also demonstrated by Mozafar (1989). Choi et al. (2015) found that insufficient B supplies reduced the Ca content of tomato fruit clusters in water culture after 36 days. Dursun et al. (2010) also investigated the effect of B fertilization on tomato, but this study focused on the elemental composition of the vegetative parts of the plant. Similarly, Eraslan et al. (2007) studied the effect of toxic amounts of B on the vegetative parts of tomatoes on soil media and showed an increase in N, P, and K contents in the plant in parallel with the decrease in biomass. Ghasemidehkordi et al. (2018) studied the B content of leafy vegetables and irrigation water in Iran but did not examine the relationship between these parameters. Lucho-Constantino et al. (2005) studied the effect of irrigation with wastewater with high B concentration in a sample area in Mexico, which showed the risk posed by B, but did not examine the direct relationship between the B content of irrigation water and the elemental composition of plants.

This study aimed to investigate the effect of B irrigation on the elemental composition and the B accumulation in the edible parts in four vegetables (i.e., tomato, potato, cabbage, and green bean) grown on soils of different textural classes. The possible toxicity effect of the treatments was monitored by measuring photosynthetic parameters. The B concentration in the irrigation water (0.1 and 0.5 mg/L) was chosen to avoid acute toxicity and to reveal the effects of B in physiologically healthy plants. These concentrations are below the B threshold values for irrigation water in most countries (Jeong et al., 2016). According to the study by Bingham (1973), potato and tomato can be considered as semi-tolerant plants of B, being able to tolerate up to 5 mg/L B in the soil solution without negative effects. Cabbage can tolerate B concentrations of up to 10 mg/L. No data appear in the studies available in the literature on the B tolerance of green bean. Wilcox (1960) suggested the critical concentrations of 1–2 and 2–4 mg B/L in the irrigation water for semi-tolerant and tolerant plants, respectively. However, these values are highly dependent on soil properties and irrigation management, which influence the availability of B.

In the study by Fernandez-Jaramillo et al. (2012), chlorophyll fluorescence (Fv/Fm) imaging and the chlorophyll content index (CCI) were used to determine the changes in photosynthetic processes, which are the indicators of plant health and B phytotoxicity, but the reports on the use of quantum efficiency of photosystem II (PSII) photochemistry to determine the toxicity of different B concentrations in tissues are still rare. Most of the studies in the literature focus on the plant physiological processes caused by B deficiency or discuss the simultaneous application of B and other microelements and macroelements (Chapman et al., 1997; Herrera-Rodríguez et al., 2010). Moreover, most of the studies were performed in a hydroponic system. The Fv/Fm ratio significantly decreased in plants due to the effect of B toxicity (Guidi et al., 2011; Landi et al., 2012). The Fv/Fm reduction could be caused by the structural damage in thylakoid membranes, changing the electron transport and inhibiting the photoassimiliation. The photoinhibition is also amplified by the reduction in stomatal conductance under B loading. The stress-induced changes in the quantity and composition of photosynthetic pigments caused variations in the optical features of leaves and plant chlorophyll content, which are the indicators of phytotoxicity (Füzy et al., 2019).

The questions raised in this study were as follows: (i) Does the B concentration of the irrigation water influence the B concentration of plants and the transfer of B from soil to plants and within the plants on different soils? (ii) Does B concentration in irrigation water influence some parameters of the photosynthetic system and biomass of plants grown on different soils? (iii) Does the B concentration of the irrigation water influence the elemental composition of the edible parts of vegetables grown on different soils?

## Materials and Methods

### Experimental Setup

The effect of irrigation with a B solution was investigated in a pot experiment conducted in a greenhouse with two open sides at the Experimental Station of the Centre for Agricultural Research in Őrbottyán, Hungary, using 10-L pots with four holes (Ø 0.5 cm) in the bottom so that leached water could flow out (Dobosy et al., 2020). The bottom of each pot was filled with a 1-cm layer of gravel (4–8 mm) covered with a fine synthetic fiber fabric on which 10 kg of soil was placed (Dobosy et al., 2020). The three topsoils (0–20 cm) investigated were as follows: sand [Mollic Umbrisol (Arenic) from Őrbottyán], silty sand (Luvic Calcic Phaeozem from Gödöllő), and silty soil (Calcic Chernozem from Hatvan). The soil properties are shown in Table 1. The total number of pots was measured as follows: three soils × two B doses (i.e., 0.1 and 0.5 mg B/L; actual measured concentrations were 0.134 and 0.506 mg B/L, respectively) + control (i.e., tap water; actual measured B concentration: 0.023 mg B/L) × 4 plant species × 3 replications = 108. The test plants were tomatoes (e.g., *Solanum lycopersicum*, cv. Mano; ZKI), green beans (e.g., *Phaseolus vulgaris*, cv. Golden Goal; Réde), potatoes (e.g., *Solanum tuberosum* L. cv. Balatoni rózsa), and cabbages (e.g., *Brassica oleracea* L. var. *capitata* cv. Zora; Réde). Before the pot experiment was set up, tomato and cabbage seeds and potato tubers were germinated and planted in propagation trays (i.e., 1 seed or tuber/cell) filled with VEGASCA Bio vegetable organic growing medium (i.e., mixture of peat and gray cattle manure compost; OM > 50%; *N* > 0.3%; P_2_O_5_ > 0.1%; K_2_O > 0.1%; pH of 6.8). The seedlings were cultivated in a random arrangement in a growth chamber for 22 days at day/night temperatures and photoperiods of 26/18°C and 16/8 h, respectively, with a photon flux density of 500 μmol/m^2^/s and relative humidity of 50–70%. The seedlings were irrigated weekly with 60-ml tap water. The soil-free seedlings were then transplanted into the experimental plastic pots (1 seedling/pot) after a 6-day acclimatization period in the greenhouse. The germinated seeds of green beans were planted directly into the experimental soils (1 seed/pot).

**Table 1:** Selected properties of the applied soils.

Properties	Sand	Silty sand	Silt
pH-H_2_O	7.96	6.83	7.34
OM (%)	0.91	1.24	2.12
CaCO_3_ (%)	1.45	0.08	0.20
CEC (Na meq/100 g)	9	17	37
Total N (%)	0.064	0.092	0.135
NH_4_-N (mg/kg)	1.4	2.3	3.9
NO_3_-N (mg/kg)	4.7	2.3	14.2
Total K (mg/kg)	3,164	7,250	7,639
AL-K_2_O (mg/kg)	74	174	176
Total P (mg/kg)	449	446	412
AL-P_2_O_5_ (mg/kg)	131	238	81
Total B (mg/kg)	2.63	5.26	9.14
Water-soluble B (mg/kg)	0.102	0.267	0.306
Total Mg (mg/kg)	4,035	3,942	5,399
Total Fe (mg/kg)	10,028	17,875	29,651
Total Cu (mg/kg)	5.00	13.8	20.1
Total Zn (mg/kg)	22.9	49.1	62.1
Clay (<0.002 mm, %)	14	23	34
Silt (0.002–0.02 mm, %)	18	30	50
Sand (0.02–2 mm, %)	69	46	16

AL, ammonium-lactate soluble; total, aqua regia-soluble.

The irrigation water was delivered using individual drip stakes placed in each pot. After planting the seedlings were irrigated with tap water for 3 weeks, but during the growing period, all the plants (including the control) were watered weekly with Hoagland solution (200 ml/pot). Irrigation with B solution (H_3_BO_3_ diluted with tap water) started 3 weeks after planting. The tap water was stored in 0.5 m^3^ tanks (i.e., separate tanks for each irrigation solution) before applying in order to reduce the chlorine concentration. The daily volume of irrigation water was adjusted to the water requirements of the plant species. A monitoring system (Sensor: Decagon EC-5) installed at a depth of 10 cm measured the soil moisture content every hour. The irrigation system delivered the set amount of water at 7 am every day. The irrigation details can be found in Table 2.

**Table 2:** Irrigation parameters in the greenhouse.

Parameters	Tomato	Green bean	Potato	Cabbage
Growing period (transplanting-harvest)	24 May–21 August	23 May–24 July	24 May–17 July	17 July–25 September
Length of growing period (days)	88	63	55	71
Total irrigation (ml/pot)	36,983	15,645	19,553	22,965
B load in 0.1 mg/L treatment (mg/pot)	2.71	0.78	1.07	1.91
B load in 0.5 mg/L treatment (mg/pot)	13.56	3.88	5.35	9.53

The experimental area received natural light in a greenhouse, where the climate data (i.e., mean day and night temperature, air humidity, and photon flux density of photosynthetically active radiation) were continuously monitored during the growing period (Table 3). Pesticides were applied as necessary.

**Table 3:** Greenhouse parameters during the growing period.

Parameters	Tomato	Green bean	Potato	Cabbage
Mean day temperature (°C)	26.6 ± 3.3	25.5 ± 3.3	25.6 ± 3.5	25.5 ± 4
Mean night temperature (°C)	19.1 ± 2.3	18.3 ± 2.3	18.1 ± 2.3	18.2 ± 3.4
Photosynthetically active radiation (μmol/m^2^/s)^∗^	982 ± 419	1,045 ± 484	1,097 ± 489	703 ± 197
Air humidity (%)	69.4 ± 8.1	70.3 ± 8.6	69.7 ± 23.3	72.2 ± 23
Soil moisture (% v/v)	22 ± 3	24 ± 3	22 ± 6	22 ± 6

∗ Spectral band: 400–700 nm.

### Chemical Analysis

The plants were harvested and cleaned with deionized water, after which the root, shoot (stem + leaf), and fruit were separated, and the fresh weight (FW) of the plant parts was measured. The shoot and root samples were dried at 40°C for 2 days in a laboratory dryer, while tomato, green bean, and potato fruit samples were freeze-dried at −70°C in Christ Alpha 1 equipment (Martin Christ Gefriertrocknungsanlagen GmbH, Germany; 200 Pa for 72 h), after which the dry mass of the samples was determined. All the samples were homogenized in a household blending machine equipped with plastic housing and a stainless-steel blade. The dried, homogenized samples were mineralized in a microwave-assisted acid digestion system (TopWave, Analytik Jena, Germany). Twelve PTFE bombs were used, 1 for the blank and 11 for the samples, and the blank was measured each time. Dried plant samples (400–500 mg) were digested in a mixture of 7 cm^3^ 67% HNO_3_ and 3 cm^3^ 30% H_2_O_2_. After digestion, the internal standards were added to the solutions and the volume was made up to 15 cm^3^ with deionized water. The concentrations of B, macroelements, and microelements were measured by inductively coupled plasma mass spectrometer (ICP-MS).

After removing plant residues, the soil samples were dried and sieved through a 2-mm size mesh. The pseudo-total B, K, and P contents of soil samples were determined after digestion with aqua regia in a microwave Teflon bomb (MSZ-21470-50, 2006). The plant-available B fraction of soil samples was measured in 0.5 M NH_4_ acetate + 0.02 M ethylenediaminetetraacetic acid (EDTA) extract (Lakanen and Erviö, 1971). The plant-available P and K contents were determined in ammonium acetate–lactate (AL) extract (AL-P_2_O_5_ and AL-K_2_O; Egnér et al., 1960). The element contents were measured using the ICP-MS method. The operating conditions of the ICP-MS instrument are summarized in Supplementary Table 1. The total N content was analyzed according to the Kjeldahl method (ISO 11261, 1995), and the mineral N (NH_4_-N and NO_3_-N) contents were measured from KCl extracts (MSZ-20135, 1999). The soil pH was measured according to the Hungarian Standard (MSZ-08-0206/2, 1978) in a soil:water suspension of 1:2.5 after mixing for 12 h. Soil OM content was determined using the modified Walkley–Black method (MSZ-08-0452, 1980). The soil CaCO_3_ content was measured by the Scheibler gas-volumetric method (MSZ-08-0206/2, 1978), and the cation exchange capacity (CEC) values were measured by the modified method of Mehlich (MSZ-08-0215, 1978).

### Translocation Coefficient

The translocation coefficient (TC) has been calculated according to Wang et al. (2017). Three steps of translocation have been defined as follows: (i) from soil to root (root/soil total B content including the B added with irrigation); (ii) from root to shoot (shoot/root B content); and (iii) from shoot to fruit (fruit/shoot B content).

### Quantum Efficiency of PSII Photochemistry and CCI

The CCI values of potato, tomato, green bean, and cabbage cultivars were measured at the harvesting stage. The CCI values of the youngest adult leaves were determined *in situ* using a CCM-200 plus Chlorophyll Content Meter (Opti-Sciences, Hudson, NY, United States) and calculated from the average of three measurements per plant. The quantum efficiency (Fv/Fm) of PSII photochemistry was measured with Os30p+ handheld chlorophyll fluorometer (Opti-Sciences). To indicate the potential stress caused in the crops by B treatment, Fv/Fm ratios were measured after 15-min dark adaptation.

### Statistical Analysis

The data were analyzed for treatment effects using factorial analysis of variance (ANOVA) and Tukey’s *post hoc* test. The variance was calculated for soil type and B concentration of the irrigation water. Significant differences between the treatments were calculated at the *p* < 0.05 level. Statistica version 13 (Statsoft Inc, Palo Alto, CA, USA) software was used for all the statistical evaluations. Data visualization was performed with R statistical software (R Core Team, 2019).

## Results

### Soil Chemical Properties

Regarding their original B contents, the applied soils can be categorized as B-poor soils as they are in the low end of the natural concentration range (Kabata-Pendias and Pendias, 2001). As a result of irrigation, the total B concentration did not change in the soils. The plant-available B content increased as a function of the B concentration in the irrigation water, but this change was only significant in the case of cabbages, green beans, and tomatoes cultivated on sandy soil (Supplementary Table 2). The pH and plant-available N, P, and K contents did not change significantly during the experiment as a function of the B concentration in the irrigation water.

### Plant Biomass

Based on the biomass values, none of the irrigation water was toxic to the plants. In the case of tomatoes and potatoes, the treatment did not influence the biomass of the plant organs (i.e., root, shoot, and fruit/tuber; Supplementary Table 3). The average FW of tomato fruit was 306 ± 77 g/plant and the dry weight (DW), 19.8 ± 4.6 g/plant. The shoot and root biomass were 26.3 ± 5.5 g and 3.23 ± 0.53 g DW, respectively. The average FW of potato tuber was 179 ± 19 g/plant and its average DW was 34.2 ± 4.4 g/plant, while the average shoot and root DW were 9.38 ± 1.07 g/plant and 2.7 ± 0.6 g/plant, respectively. Neither FW nor DW of the green bean fruits nor the shoot DW (100 ± 23 g/plant, 10.6 ± 3 g/plant, and 12.3 ± 1.8 g/plant, respectively) changed as a function of B treatment on any soil. The dry biomass of green bean roots was also unaffected by B irrigation, being 4.1 ± 0.8 g/plant on sandy soil and 1.84 ± 0.17 g/plant on the other two soils. The irrigation and soil-type combinations did not modify the biomass of cabbage leaves (520 ± 48 g FW/plant and 53 ± 8.4 g DW/plant), but the root DW was increased from the control value of 1.95 g/plant to 4.50 g/plant, applying 0.5 mg B/L irrigation water on sandy soil.

### Quantum Efficiency of PSII Photochemistry and CCI

No visible toxic symptoms were observed on B-irrigated plants compared with the control plants (Table 4). The Fv/Fm ratio of potato, cabbage, and tomato leaves ranged from 0.667 to 0.83, which was not significantly affected by either the soil type or the B treatment. The lowest Fv/Fm values were measured in the control potato cultivated on sand and the highest values were measured in cabbages grown on silty soil in the control treatment.

**Table 4:** Chlorophyll content index (CCI) and quantum efficiency of PSII photochemistry (Fv/Fm) of investigated plants.

Soil	Irrigation water	Tomato		Cabbage		Green bean		Potato	
		Fv/Fm	CCI	Fv/Fm	CCI	Fv/Fm	CCI	Fv/Fm	CCI
Sand	Control	0.786 ± 0.009a	12.5 ± 3.1ab	0.831 ± 0.003a	20.9 ± 4.3a	0.735 ± 0.012a	15.5 ± 4.6ab	0.667 ± 0.028a	11.1 ± 1.0a
	0.1 mg B/L	0.780 ± 0.010a	15.6 ± 4.2abc	0.829 ± 0.004a	24.4 ± 7.3a	0.723 ± 0.004a	11.7 ± 2.1a	0.685 ± 0.018a	14.7 ± 3.6ab
	0.5 mg B/L	0.783 ± 0.004a	15.9 ± 1.8abc	0.823 ± 0.016a	31.2 ± 11.6a	0.753 ± 0.015a	16.4 ± 1.6ab	0.718 ± 0.055a	14.2 ± 2.5ab
Silty sand	Control	0.792 ± 0.006a	10.9 ± 5.1abc	0.826 ± 0.012a	24.4 ± 7.2a	0.764 ± 0.033a	20.6 ± 1.5b	0.734 ± 0.045a	13.1 ± 2.2a
	0.1 mg B/L	0.789 ± 0.006a	18.5 ± 5.1abcd	0.825 ± 0.004a	22.4 ± 5.9a	0.787 ± 0.045a	19.0 ± 2.4ab	0.738 ± 0.052a	12.1 ± 1.8a
	0.5 mg B/L	0.794 ± 0.005a	30.2 ± 6.5cd	0.822 ± 0.009a	27.9 ± 11.7a	0.794 ± 0.025a	20.8 ± 1.4b	0.725 ± 0.035a	10.9 ± 0.3a
Silt	Control	0.771 ± 0.025a	16.0 ± 8.5abc	0.835 ± 0.005a	33.0 ± 16.4a	0.773 ± 0.023a	19.1 ± 4.1ab	0.726 ± 0.046a	19.0 ± 2.0b
	0.1 mg B/L	0.791 ± 0.011a	27.5 ± 9.6bcd	0.822 ± 0.010a	25.4 ± 3.5a	0.756 ± 0.039a	18.1 ± 2.7ab	0.736 ± 0.031a	25.9 ± 1.0c
	0.5 mg B/L	0.793 ± 0.012a	33.6 ± 4.0d	0.822 ± 0.011a	30.9 ± 12.6a	0.759 ± 0.039a	16.6 ± 2.5ab	0.706 ± 0.041a	24.6 ± 0.9c

The data are given as mean ± SD of the replicates. Different letters (a–d) indicate significant differences between rows (*p* < 0.05).

Chlorophyll content index was proved to be a more sensitive indicator of the effect of B treatments than Fv/Fm. In comparison with the control, both doses of B significantly increased the CCI value in potatoes on silty soil. Potatoes had significantly higher CCI on silt than on silty sand or sand. On silty soil, the 0.5 mg/L B treatment caused a significant increase in the CCI value of tomato compared with the control. The CCI of cabbage leaves was not affected either by different B doses or by soil type. In the case of green beans, the highest CCI values were observed on silty sand.

### Element Content in Edible Parts

The boron treatment had a significant effect on the elemental composition of the edible part of the plants. The B content in the edible parts increased in all the plant species as a result of increasing B concentration in the irrigation water, but in most cases, this change was not significant. The soil type influenced the effect of B irrigation on the element content of edible parts.

**Figure 1:**
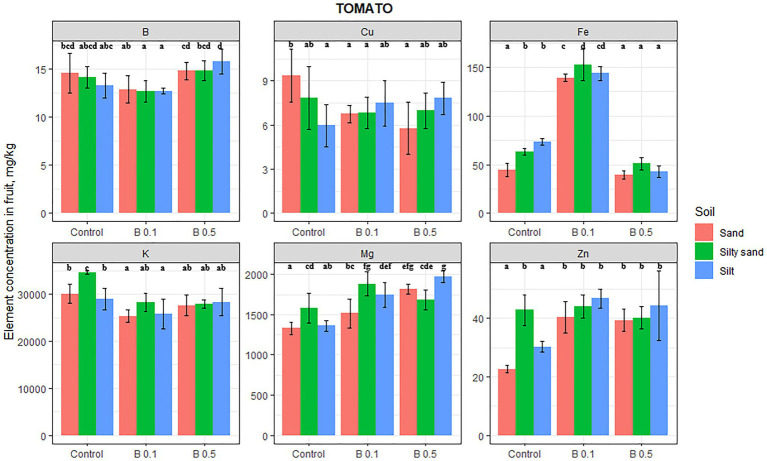
Element contents in tomato fruit in different irrigation water treatments on sand, silty sand, and silty soil (mg/kg DW). The data are means of the replicates. Different letters (a–g) indicate significant differences between treatments (*p* < 0.05).

In the case of tomatoes, irrigation with B-rich water affected the content of all the elements tested, though the B content of fruit increased significantly (by 18%) only on silty soil in the 0.5 mg B/L treatment (Figure 1). On sand and silt substrate, the Mg content in the tomato fruit increased by the B concentration of irrigation water, being 36% higher than the control in the 0.5 mg B/L treatment on sand and 44% higher on silty soil. Only the K content of tomato changed on sand and silty sand as a result of the B treatments, with average decreases of 12 and 18% compared with the control. The Fe content of tomato fruit tripled on sandy soil and doubled on silty sand and silty soil in the 0.1 mg B/L treatment. However, in the 0.5 mg B/L treatments, the Fe content remained at the control level on sandy soil, while it decreased significantly on silty sand and silty soil compared with the control. The Cu content of tomato fruit was only affected by B irrigation on sandy soil, where the Cu content diminished by 33% on average. In contrast, the Zn content in tomato fruit increased significantly in the B treatments on sandy and silty soil by 75 and 50%, respectively. Tomato was the only vegetable studied whose Zn content was increased by the B treatment.

**Figure 2:**
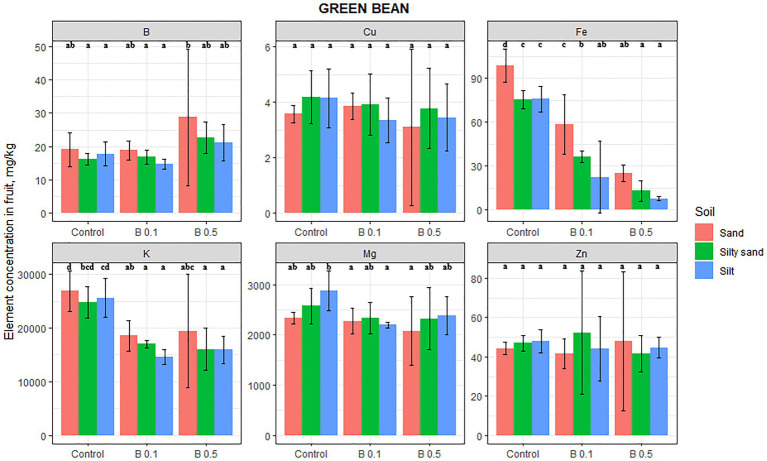
Element contents in green bean fruit in different irrigation water treatments on sand, silty sand, and silty soil (mg/kg DW). The data are means of the replicates. Different letters (a–d) indicate significant differences between treatments (*p* < 0.05).

The B content in green beans increased as a function of the B treatments, but this change was not significant (Figure 2). Significant changes were recorded only after B irrigation in Mg, K, and Fe. The Mg content decreased on all the soils, but the change was significant only in the 0.1 mg B/L treatment on silty soil (24%). The K content decreased significantly on every soil as a result of B treatment, but there was no difference between the effect of the two B doses, the reduction being 29, 33, and 40% compared with the control on sand, silty sand, and silty soil, respectively. The Fe content in green beans was also negatively affected by B irrigation, decreasing by 75, 83, and 90% on sand, silty sand, and silty soil, respectively, in the 0.5 mg B/L water treatment compared with the control. The Cu and Zn contents of green beans were unaffected by the B treatments.

**Figure 3:**
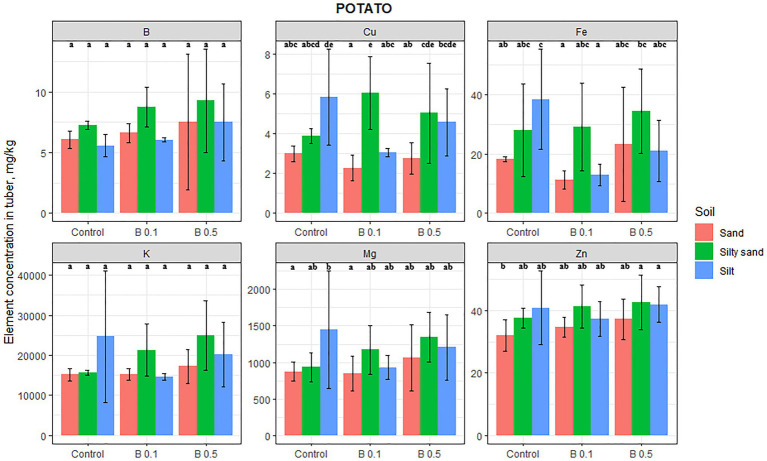
Element contents in potato tuber in different irrigation water treatments on sand, silty sand, and silty soil (mg/kg DW). The data are means of the replicates. Different letters (a–e) indicate significant differences between treatments (*p* < 0.05).

Among the species tested, the elemental composition in the edible part of potatoes was the least affected by B irrigation (Figure 3). The B content in potato tubers increased as a function of B treatment on every soil, but this change was not significant. Similarly, Mg and K contents also increased on lighter textured soils, but this change was not significant either. B treatment had a negative effect on the Fe content on silty soil, with an average decrease of 56%. The only other element significantly influenced by the B treatment was Cu, which exhibited a 55% increase in the 0.1 mg B/L treatment on silty sand compared with the control. There was no change in the Zn content of potato tuber as a function of B treatments.

**Figure 4:**
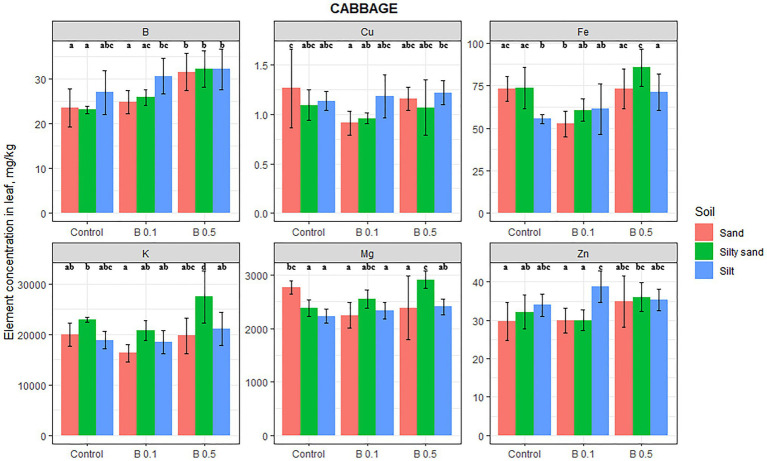
Element contents in cabbage leaf in different irrigation water treatments on sand, silty sand, and silty soil (mg/kg DW). The data are means of the replicates. Different letters (a–d) indicate significant differences between treatments (*p* < 0.05).

Boron treatment significantly influenced the B, Mg, K, Fe, and Cu contents of cabbage leaves (Figure 4), but the changes were highly dependent on the soil type. The concentration of B of the irrigation water influenced the elemental composition to the greatest extent on sand and silty sand. B, Mg, Fe, and Cu were influenced by B treatment on sandy soil, B, Mg, and K on silty sand, and only Fe on silty soil.

The increase in B content of cabbage leaves in the 0.5 mg B/L treatments was 34 and 39% on sand and silty sand, respectively. An increase of 19% was also observed on silty soil, but this was not significant. On sandy soil, B treatment significantly decreased the Mg content of cabbage leaves (by 14%), while on silty sand and silt, the opposite trend was observed, with a 22 and 8% increase in the 0.5 mg B/L treatment compared with the control, respectively. Although this change in the silty soil was not statistically significant, only the K content in the leaves increased by 20% on silty sand in the 0.5 mg B/L water treatment compared with the control. The K content also increased on silty soil as a function of the B treatment, but the 12% change was not significant. The sign of the change in Fe content varied with the soil type. On sandy soil, it decreased by 28% in the 0.1 mg B/L treatment, but on silty sand, it increased by 30% in the 0.5 mg B/L treatment compared with the control. There was a significant decrease of 28% in the Cu content of cabbage leaves as a result of the 0.1 mg B/L treatment, but Zn was unaffected by the B irrigation on any of the soils.

### Boron Translocation

The root/soil B translocation was higher on sandy soil (3.8 on average) than on silty sand (2 on average) and silt (1.4 on average) for all the plant species (Table 5). If TC is higher than 1, it means that B accumulates in the given plant part (Sasmaz, 2008). In the case of green beans, the B concentration in the irrigation water did not influence the root/soil TC on any soil. The B translocation to cabbage roots was significantly higher in the 0.1 mg B/L water treatment (2.9) than in the control (2.2) or the 0.5 mg B/L water treatment (2.0) on silty sand. In the case of tomatoes, the 0.1 mg B/L concentration resulted in the lowest root/soil TC on all three soils (1.4 on average). Potatoes had the highest root/soil TC values on average (3.1). The B treatment caused only a significant increase in the potato root/soil TC on sandy soil, from 3.9 (control) to an average value of 5.1 in the B treatments.

**Table 5:** Translocation coefficients of B between soil and plant and between different plant parts.

Soil	Irrigation water	Root/Soil				Shoot/Root				Fruit/Shoot		
		Tomato	Green bean	Potato	Cabbage	Tomato	Green bean	Potato	Cabbage	Tomato	Green bean	Potato
Sand	Control	3.02 ± 0.10f	3.98 ± 0.29b	3.89 ± 0.16d	4.22 ± 0.07b	2.74 ± 0.05b	1.15 ± 0.14c	2.52 ± 0.32a	1.05 ± 0.09ab	0.33 ± 0.04a	0.79 ± 0.15b	0.12 ± 0.01a
	0.1 mg B/L	2.27 ± 0.12d	4.02 ± 0.85b	5.23 ± 0.07c	4.22 ± 0.14b	4.37 ± 0.20c	1.35 ± 0.25ac	2.21 ± 0.20a	1.07 ± 0.05ab	0.23 ± 0.01ab	0.66 ± 0.06ab	0.11 ± 0.01a
	0.5 mg B/L	2.77 ± 0.17e	3.41 ± 0.42b	4.92 ± 0.37c	3.75 ± 0.35b	3.58 ± 0.33a	2.70 ± 0.29de	2.40 ± 0.59a	1.35 ± 0.20bc	0.23 ± 0.01ab	0.53 ± 0.08ab	0.11 ± 0.04a
Silty sand	Control	1.69 ± 0.04c	1.82 ± 0.14a	2.70 ± 0.12b	2.21 ± 0.18d	3.05 ± 0.10b	1.52 ± 0.16abc	2.99 ± 0.24ab	1.06 ± 0.10ab	0.28 ± 0.02ab	0.60 ± 0.05ab	0.09 ± 0.01a
	0.1 mg B/L	1.15 ± 0.06ab	1.59 ± 0.23a	2.64 ± 0.20b	2.86 ± 0.15e	3.93 ± 0.10ac	2.11 ± 0.26bde	3.22 ± 0.16ab	0.90 ± 0.07a	0.28 ± 0.01ab	0.51 ± 0.04a	0.10 ± 0.01a
	0.5 mg B/L	1.47 ± 0.03c	1.41 ± 0.06a	2.84 ± 0.30b	1.97 ± 0.20cd	3.88 ± 0.11a	2.76 ± 0.05e	3.91 ± 0.77b	1.52 ± 0.18cd	0.15 ± 0.13b	0.57 ± 0.05ab	0.08 ± 0.02a
Silt	Control	1.18 ± 0.07b	1.44 ± 0.24a	1.75 ± 0.04a	1.29 ± 0.05a	2.79 ± 0.09b	1.53 ± 0.29abc	2.80 ± 0.47ab	1.60 ± 0.18cd	0.31 ± 0.02a	0.64 ± 0.14ab	0.09 ± 0.02a
	0.1 mg B/L	0.93 ± 0.01a	1.29 ± 0.16a	1.83 ± 0.41a	1.50 ± 0.12ac	3.74 ± 0.13a	1.84 ± 0.40ab	2.91 ± 0.59ab	1.53 ± 0.05cd	0.27 ± 0.01ab	0.49 ± 0.12a	0.09 ± 0.01a
	0.5 mg B/L	1.14 ± 0.05ab	1.38 ± 0.11a	1.86 ± 0.04a	1.27 ± 0.04a	3.99 ± 0.17ac	2.03 ± 0.11abd	3.38 ± 0.35ab	1.80 ± 0.13d	0.24 ± 0.01ab	0.56 ± 0.08ab	0.09 ± 0.02a

The data are given as means ± SD of the replicates. Different letters (a–f) indicate significant differences between rows (treatments: soil and irrigation water; *p* < 0.05).

The soil type did not influence the shoot/root TC value, which was higher than 1 for all the species, indicating the accumulation of B in the shoot. The shoot/root TC values increased as a function of the B concentration in the irrigation water for all the species on all the soils, but this change was not always significant. The shoot/root TC for tomatoes was 37% higher on average in the B treatments than in the control on every soil. For green bean plants, the shoot/root TC significantly increased as a result of the 0.5 mg B/L treatment compared with the control on sand (2.8 times) and silty sand (1.8 times). For potatoes, the B concentration of the irrigation water did not cause any difference in the B translocation from root to shoot. In the case of cabbages, the 0.5 mg B/L treatment resulted in 1.4 times higher shoot/root TC compared with the control on silty sand, but on other soils, there was no significant increase. Based on the average shoot/root TC values, all three plant species can be considered as B accumulators. Tomatoes accumulated the most B in the shoot compared with the root, followed by potatoes, green beans, and cabbages. The average transfer values were 3.6, 2.9, 1.9, and 1.3, respectively.

The fruit/shoot TC values were below 1, showing that B was not accumulated in the edible parts of tomatoes, potatoes, and green beans.

## Discussion

### Biomass

Boron may either increase or decrease plant yield depending on the circumstances. In this study, the B irrigation had no toxic effect on the plants under the given conditions. One specific symptom of B toxicity is a decrease in leaf and root biomass (Roessner et al., 2006; Turan et al., 2009), which was not observed in this experiment. On B-poor soils B fertilization has been reported to increase plant yield (Sarkar et al., 2018), but this was not confirmed in this study probably due to the low amount of the B applied (Singh and Singh, 1990; Ganie et al., 2014).

### Photosynthetic Efficiency and CCI

The high concentrations of B in the plant growth medium or in the irrigation water can result in oxidative damage induced by antioxidant enzyme activity. The maximum quantum yield of Fv/Fm significantly decreased in plants exposed to B toxicity (Landi et al., 2012). Guidi et al. (2011) reported that a B concentration of up to 2 mg/L in the irrigation water caused no change in the Fv/Fm values of tomato leaves. When B stress levels were assessed, the chlorophyll fluorescence was found to be inversely proportional to B toxicity in potato cultivars (Ayvaz et al., 2016). In this study, the B treatments did not have a significant effect on Fv/Fm values (Table 4). The optimal Fv/Fm value of all plant species is around 0.8 (Pang et al., 2004). In this experiment, the minimum Fv/Fm values, indicating photoinhibition (0.667–0.718), were observed in the case of potatoes grown on sand.

The CCI values showed no change as a function of B treatments for green beans and cabbages. According to the study of Bingham (1973), cabbages tolerate B without symptoms of phytotoxicity. However, Pandey and Archana (2013) demonstrated that higher B doses (3.3 and 33 mg B/L) may reduce chlorophylls a and b and carotenoids in cabbage leaves. The high concentrations of B (2 and 4 mg B/L) reduced the dry matter, fruit yield, and chlorophyll content of tomatoes (Kaya et al., 2009). In this study, the 0.5 mg/L dose of B had a beneficial effect on the CCI-based chlorophyll content in tomato leaves, which could be attributed to the effect of B on the uptake of microelements, especially Mg (Bohn et al., 2004). The plant physiological measurements indicate that green beans are sensitive to B, which is required for nodule formation and N_2_-fixation processes in legumes and is essential for *Rhizobium*-legume signaling and nod-gene expression (Bolanos et al., 1994; Redondo-Nieto et al., 2001). Averaged over each species, the highest CCI values resulted on silty soil, possibly due to the higher concentration of N (Table 1). A close correlation has been reported between leaf chlorophyll content and plant N status in many agricultural crops (Liu et al., 2019; Padilla et al., 2019).

### Element Contents

The B content of the vegetables remained in the range of values suitable for human consumption even after the treatments. For the required minimum daily intake (1 mg B/day), either 520 g of green beans, 370 g of cabbages, 770 g of potatoes, or 1,110 g of tomatoes should be consumed. In silty soil, a moderate B increment was observed in tomato fruit. Dursun et al. (2010) also reported a significant 7-fold increment of B in tomato fruit after B fertilization with 0.5 mg/kg soil. The element concentrations measured in tomato fruit were within the range reported by Kelly and Bateman (2010), with the exception of Fe in the 0.1 mg B/L treatment. However, Demir et al. (2010) measured similar Fe concentrations in tomatoes grown on clay loam soil. The Fe content of tomato fruit and leaves changes during the ripening process. In the final stage of fruit ripening, the Fe content increases, so the higher Fe content can indicate more ripped fruit (Chohura et al., 2009; Ramesh et al., 2020). According to this, the 0.1 mg B/L dose may have promoted the ripening process, which is shown by the higher Fe content on each soil in this treatment. However, the 0.5 mg B/L treatment on the sandy soil did not affect the ripening process (i.e., Fe content is equal to the control), while, on the other two soils, the lower Fe content compared with the control may indicate inhibited fruit maturation. The increase in Mg content of tomato fruit as a result of B treatment is consistent with the changes in the vegetative parts of tomatoes observed by Eraslan et al. (2007), but contradictory with the results observed by Dursun et al. (2010). The higher Mg content is favorable, since the presence of sufficient Mg in the human diet reduces the risk of cancer (Gile et al., 2020). The Cu content of tomato fruit was in the same range as detected on non-contaminated soil by Ginocchio et al. (2002), who concluded that the Cu content of tomato fruit is determined by the soil Cu content, pH, and CEC. Since these values were not modified by the irrigation water in this experiment, the decrease in the Cu content on sandy soil could be explained by the effect of B addition. To the best of our knowledge, no information is available about the effect of B on the Cu content of tomato fruit, although B fertilization resulted in higher Cu levels in the vegetative parts of tomato ffruit in the experiment performed by Dursun et al. (2010). The results of this study indicate that the application of B may increase the Zn content of tomato fruit. This effect of B has only been demonstrated in the studies in the literature for tomato shoots (Güneş et al., 1999; Dursun et al., 2010). An increase in the Zn content of tomato fruit would be beneficial, since over 25% of the world population lives with the risk of Zn deficiency (Maret and Sandstead, 2006).

Based on the elemental composition, B treatment decreased the nutritional value of green beans on all three soils. According to the studies by Singh and Singh (1990) and Ganie et al. (2014), the optimal B fertilization of green bean is about 1 kg/ha. This value represents about 3 mg/kg content in the plowed layer, which is about 10 times higher than the B dose in the 0.5 mg B/L treatment (Table 2). The B content recorded in green beans in the 0.5 mg B/L treatment can be categorized as high, since it exceeds the range reported by Beebe et al. (2000) based on 1,000 samples (maximum value: 18 mg/kg). The decrease in the K content of green beans fruit contradicts the results suggested by Ganie et al. (2014), who reported an increase in K content after 0.5–1.5 kg/ha B fertilization. The decrease in the K content is unfavorable, because the recommended intake of K is about four times higher than the average consumption of this element in the current diet (Sebastian et al., 2006). The Fe content decreased to half the average value for beans in 0.5 mg B/L treatment, which is unfavorable from a human nutrition point of view, as Fe deficiency is one of the most common health issues in the world (Beebe et al., 2000; Blanco-Rojo and Vaquero, 2019). No results are available on the effect of B fertilization on the Mg content of green beans, but similar decreases in Mg content have been observed in the vegetative parts of tomatoes, cucumbers, peppers, and tobacco after the application of B to the soil (López-Lefebre et al., 2002; Dursun et al., 2010).

The B treatment had less effect on the elemental composition of potato tubers, which may vary greatly depending on the growth conditions such as soil properties, fertilization, and irrigation, and such variation could not be observed in this experiment (White et al., 2009). Even after the changes caused by the application of B, the Fe and Cu contents of potato tubers were still in the range reported by True et al. (1978).

Among the vegetables studied, the response of cabbages to the B treatment showed the most significant differences as a function of soil type. Although B irrigation influenced the B, Mg, K, Fe, and Cu contents, there was no element on which B had a significant effect on all three soils. The application of B only led to a decrease in the nutritional value of cabbage on sandy soil by diminishing the Fe, Cu, and Mg contents. The vegetables from the cruciferous family like cabbage have high B requirements, which explains the increasing B content of cabbage leaves as a function of the B treatment (Choi et al., 2016). According to the ranking suggested by Tariq and Mott (2007), the B content in cabbages can be considered as adequate in all the treatments. Even after the changes caused by the addition of B, the Mg content of cabbages always remained in the range reported by Hara and Sonoda (1981).

### Translocation

The soil type influenced the root/soil TC, but it had no effect on the distribution of B (i.e., shoot/root and fruit/shoot TC) in plants. The lighter the soil texture, the more B was absorbed by the roots from irrigation water, which is in accordance with the results suggested by Wear and Patterson (1962). The uptake of B is more influenced by the B content in the soil solution than by the B adsorbed on soil particles (Keren et al., 1985). In the case of adequate B supply, the uptake mechanism is passive diffusion (Dannel et al., 2002). Thus, the higher uptake on sandy soil can be explained by the lower CEC of this soil and the relatively high amount of added B compared with the original B content of the soil.

Within the plants, B mostly moves passively in the xylem *via* the transpiration stream (Landi et al., 2013). Each of the species investigated was able to absorb B from the soil and store it in aboveground parts (Sasmaz, 2008). According to the findings suggested by Choi et al. (2015), the tomato organs that accumulate the most B are the stem and leaves, which is in accordance with the results of this study.

## Conclusion

The main conclusion can be summarized as follows:

- The B treatment had no effect on the biomass of the edible parts of tomatoes, green beans, potatoes, and cabbages but modified the nutritional value of these vegetables by changing their element contents.
- These changes were influenced by soil type.
- The B content showed a significant increase only in the case of tomatoes grown on silty soil and cabbages grown on light-textured soils irrigated with water containing 0.5 mg/L B.
- The B irrigation had the most significant effect on the elemental composition of tomato fruit, as it affected the B, Mg, K, Fe, Cu, and Zn contents.
- The irrigation water containing 0.1 mg/L B promoted the ripening of tomatoes on all the soils.
- The B treatment decreased the nutritional value of green beans by diminishing its K and Fe contents on all the soils.
- The element content of potatoes was almost unaffected by the B treatments.
- The nutritional value of cabbages was modified only on sandy soil, where the Mg, Fe, and Cu contents of the leaves decreased as a result of the B treatments.

The chlorophyll content index was proved to be a more effective parameter for the indication of the effects of the B irrigation than Fv/Fm. The B treatments applied in this study caused no stress to the plants, and in the case of tomatoes and potatoes, an increase in chlorophyll content was observed on silty soil.

## Data Availability Statement

The raw data supporting the conclusions of this article will be made available by the authors, without undue reservation.

## Author Contributions

MR contributed to the design and implementation of the experiment and prepared the manuscript. PR contributed to the implementation of the experiment and conducted the statistical data analyses. AF conducted the statistical data analyses and proofread the manuscript. NU contributed to the data evaluation and proofread the manuscript. PD was the leader of the project, performed the analytical experimental work, and proofread the manuscript. GZ provided substantial contributions to the conception of the study and proofread the manuscript. NS-V contributed to the implementation of the experiment. AM performed the soil analysis. TT carried out the photosynthetic efficiency and chlorophyll content measurements and prepared and proofread the manuscript. All authors contributed to the article and approved the submitted version.

### Conflict of Interest

The authors declare that the research was conducted in the absence of any commercial or financial relationships that could be construed as a potential conflict of interest.

## References

[ref1] Ayvaz M. Guven A. Blokhina O. Fagerstedt K. V. (2016). Boron stress, oxidative damage and antioxidant protection in potato cultivars (*Solanum tuberosum* L.). Acta Agric. Scand. Sec. B Soil Plant Sci. 66, 302–316. 10.1080/09064710.2015.1109133

[ref2] Beebe S. Gonzalez A. V. Rengifo J. (2000). Research on trace minerals in the common bean. Food Nutr. Bull. 21, 387–391. 10.1177/156482650002100408

[ref3] Berger K. C. Truog E. (1939). Boron determination in soils and plants. Ind. Eng. Chem. Anal. Ed. 11, 540–545. 10.1021/ac50138a007

[ref4] Bingham F. T. (1973). “Boron in cultivated soils and irrigation waters,” in Trace Elements in the Environment. ed. Kothnz E. L. (Washington, DC, USA: American Chemical Society), 130–138.

[ref5] Blanco-Rojo R. Vaquero M. P. (2019). Iron bioavailability from food fortification to precision nutrition: a review. Innov. Food Sci. Emerg. Technol. 51, 126–138. 10.1016/j.ifset.2018.04.015

[ref6] Bohn T. Walczyk T. Leisibach S. Hurrell R. F. (2004). Chlorophyll-bound magnesium in commonly consumed vegetables and fruits: relevance to magnesium nutrition. J. Food Sci. 69, S347–S350. 10.1111/j.1365-2621.2004.tb09947.x

[ref7] Bolanos L. Esteban E. de Lorenzo C. Fernandez-Pascual M. de Felipe M. R. Garate A.. (1994). Essentiality of boron for symbiotic dinitrogen fixation in Pea (*Pisum sativum*) rhizobium nodules. Plant Physiol. 104, 85–90. 10.1104/pp.104.1.85, PMID: 12232064 PMC159165

[ref8] Bolaños L. Lukaszewski K. Bonilla I. Blevins D. (2004). Why boron? Plant Physiol. Biochem. 42, 907–912. 10.1016/j.plaphy.2004.11.002, PMID: 15694285

[ref9] Chapman V. J. Edwards D. G. Blamey F. P. C. Asher C. J. (1997). “Challenging the dogma of a narrow supply range between deficiency and toxicity of boron,” in Boron in Soils and Plants: Developments in Plant and Soil Sciences. eds. Bell R. W. Rerkasem B. (Dordrecht: Springer), 151–155.

[ref10] Chohura P. Kolota E. Komosa A. (2009). Effect of fertilization with Fe chelates on the state of iron nutrition of greenhouse tomato. J. Elem. 14, 657–664. 10.5601/jelem.2009.14.4.657-664

[ref11] Choi E. Y. Jeon Y. A. Choi K. Y. Stangoulis J. (2016). Physiological and morphological responses to boron deficient Chinese cabbage. Hortic. Environ. Biotechnol. 57, 355–363. 10.1007/s13580-016-0023-y

[ref12] Choi E. Y. Park H. I. Ju J. H. Yoon Y. H. (2015). Boron availability alters its distribution in plant parts of tomato. Hortic. Environ. Biotechnol. 56, 145–151. 10.1007/s13580-015-0044-y

[ref13] Dannel F. Pfeffer H. Römheld V. (2002). Update on boron in higher plants ‐ uptake, primary translocation and compartmentation. Plant Biol. 4, 193–204. 10.1055/s-2002-25730

[ref14] Demir K. Sahin O. Kadioglu Y. K. Pilbeam D. J. Gunes A. (2010). Essential and non-essential element composition of tomato plants fertilized with poultry manure. Sci. Hortic. 127, 16–22. 10.1016/j.scienta.2010.08.009

[ref15] Dobosy P. Endrédi A. Sandil S. Vetési V. Rékási M. Takács T.. (2020). Biofortification of potato and carrot with iodine by applying different soils and irrigation with iodine-containing water. Front. Plant Sci. 11:593047. 10.3389/fpls.2020.593047, PMID: 33362822 PMC7755595

[ref16] Dursun A. Turan M. Ekinci M. Gunes A. Ataoglu N. Esringü A.. (2010). Effects of boron fertilizer on tomato, pepper, and cucumber yields and chemical composition. Commun. Soil Sci. Plant Anal. 41, 1576–1593. 10.1080/00103624.2010.485238

[ref17] EFSA (2018). Overview on Tolerable Upper Intake Levels as derived by the Scientific Committee on Food (SCF) and the EFSA Panel on Dietetic Products, Nutrition and Allergies (NDA). European Food Safety Authority.

[ref18] Eggert K. von Wirén N. (2016). The role of boron nutrition in seed vigour of oilseed rape (*Brassica napus* L.). Plant Soil 402, 63–76. 10.1007/s11104-015-2765-1

[ref19] Egnér H. Riehm H. Domingo W. R. (1960). Untersuchungen über die chemische Bodenanalyse als Grundlage für die Beurteilung des Nährstoffzustandes der Böden. II. Chemische Extraktionsmethoden zur Phosphor‐ und Kaliunibestimmung. Kungl. Lantbrukshögskolans Annaler 26, 199–215.

[ref20] Eraslan F. Inal A. Gunes A. Alpaslan M. (2007). Boron toxicity alters nitrate reductase activity, proline accumulation, membrane permeability, and mineral constituents of tomato and pepper plants. J. Plant Nutr. 30, 981–994. 10.1080/15226510701373221

[ref21] Fernandez-Jaramillo A. A. Duarte-Galvan C. Contreras-Medina L. M. Torres-Pacheco I. Romero-Troncoso R. D. J. Guevara-Gonzalez R. G.. (2012). Instrumentation in developing chlorophyll fluorescence biosensing: a review. Sensors 12, 11853–11869. 10.3390/s120911853, PMID: 23112686 PMC3478813

[ref22] Füzy A. Kovács R. Cseresnyés I. Parádi I. Szili-Kovács T. Kelemen B.. (2019). Selection of plant physiological parameters to detect stress effects in pot experiments using principal component analysis. Acta Physiol. Plant. 41, 1–10. 10.1007/s11738-019-2842-9

[ref23] Ganie M. A. Akhter F. Najar G. R. Bhat M. A. Mahdi S. S. (2014). Influence of sulphur and boron supply on nutrient content and uptake of French bean (*Phaseolus vulgaris* L.) under inceptisols of North Kashmir. Afr. J. Agric. Res. 9, 230–239.

[ref24] Ghasemidehkordi B. Malekirad A. A. Nazem H. Fazilati M. Salavati H. Rezaei M. (2018). Arsenic and boron levels in irrigation water, soil, and green leafy vegetables. Int. J. Veg. Sci. 24, 115–121. 10.1080/19315260.2017.1356896

[ref25] Gile J. Ruan G. Abeykoon J. McMahon M. M. Witzig T. (2020). Magnesium: the overlooked electrolyte in blood cancers? Blood Rev. 44:100676. 10.1016/j.blre.2020.100676, PMID: 32229066

[ref26] Ginocchio R. Rodríguez P. H. Badilla-Ohlbaum R. Allen H. E. Lagos G. E. (2002). Effect of soil copper content and pH on copper uptake of selected vegetables grown under controlled conditions. Environ. Toxicol. Chem. 21, 1736–1744. 10.1002/etc.5620210828, PMID: 12152777

[ref27] Goldberg S. (1997). Reactions of boron with soils. Plant Soil 193, 35–48. 10.1023/A:1004203723343

[ref28] Guidi L. Degl’Innocenti E. Carmassi G. Massa D. Pardossi A. (2011). Effects of boron on leaf chlorophyll fluorescence of greenhouse tomato grown with saline water. Environ. Exp. Bot. 73, 57–63. 10.1016/j.envexpbot.2010.09.017

[ref29] Güneş A. Alpaslan M. Çikili Y. Özcan H. (1999). Effect of zinc on the alleviation of boron toxicity in tomato. J. Plant Nutr. 22, 1061–1068. 10.1080/01904169909365695

[ref30] Hara T. Sonoda Y. (1981). The role of macronutrients in cabbage-head formation. Soil Sci. Plant Nutr. 27, 45–54. 10.1080/00380768.1981.10431253

[ref31] Herrera-Rodríguez M. B. González-Fontes A. Rexach J. Camacho-Cristóbal J. J. Maldonado J. M. Navarro-Gochicoa M. T. (2010). Role of boron in vascular plants and response mechanisms to boron stresses. Plant Stress 4, 115–122.

[ref32] Howe P. D. (1998). A review of boron effects in the environment. Biol. Trace Elem. Res. 66, 153–166. 10.1007/BF02783135, PMID: 10050917

[ref33] ISO 11261 (1995). Soil quality ‐ Determination of total nitrogen ‐ Modified Kjeldahl method. International Organization for Standardization.

[ref34] Jeong H. Kim H. Jang T. (2016). Irrigation water quality standards for indirect wastewater reuse in agriculture: a contribution toward sustainable wastewater reuse in South Korea. Water 8:169. 10.3390/w8040169

[ref35] Kabata-Pendias A. Pendias H. (2001). Trace Elements in Soils and Plants. Boca Raton: CRC Press.

[ref36] Kaya C. Tuna A. L. Dikilitas M. Ashraf M. Koskeroglu S. Guneri M. (2009). Supplementary phosphorus can alleviate boron toxicity in tomato. Sci. Hort. 121, 284–288. 10.1016/j.scienta.2009.02.011

[ref37] Kelly S. D. Bateman A. S. (2010). Comparison of mineral concentrations in commercially grown organic and conventional crops – tomatoes (*Lycopersicon esculentum*) and lettuces (*Lactuca sativa*). Food Chem. 119, 738–745. 10.1016/j.foodchem.2009.07.022

[ref38] Keren R. Bingham F. T. Rhoades J. D. (1985). Plant uptake of boron as affected by boron distribution between liquid and solid phases in soil. Soil Sci. Soc. Am. J. 49, 297–302. 10.2136/sssaj1985.03615995004900020004x

[ref39] Lakanen E. Erviö R. (1971). A comparison of eight extractants for the determination of plant available micronutrients in soils. Acta Agric. Fenn. 123, 223–232.

[ref40] Landi M. Degl’Innocenti E. Pardossi A. Guidi L. (2012). Antioxidant and photosynthetic responses in plants under boron toxicity: a review. Am. J. Agric. Biol. Sci. 7, 255–270. 10.3844/ajabssp.2012.255.270

[ref41] Landi M. Remorini D. Pardossi A. Guidi L. (2013). Boron excess affects photosynthesis and antioxidant apparatus of greenhouse *Cucurbita pepo* and *Cucumis sativus*. J. Plant Res. 126, 775–786. 10.1007/s10265-013-0575-1, PMID: 23779070

[ref42] Liu C. Liu Y. Lu Y. Liao Y. Nie J. Yuan X.. (2019). Use of a leaf chlorophyll content index to improve the prediction of above-ground biomass and productivity. PeerJ 6:e6240. 10.7717/peerj.6240, PMID: 30648006 PMC6330949

[ref43] López-Lefebre L. R. Rivero R. M. García P. C. Sánchez E. Ruiz J. M. Romero L. (2002). Boron effect on mineral nutrients of tobacco. J. Plant Nutr. 25, 509–522. 10.1081/PLN-120003379

[ref80] Lucho-Constantino C. A. Prieto-García F. Del Razo L. M. Rodríguez-Vázquez R. Poggi-Varaldo H. M. (2005). Chemical fractionation of boron and heavy metals in soils irrigated with wastewater in central Mexico. Agric. Ecosyst. Environ. 108, 57–71. 10.1016/j.agee.2004.12.013

[ref44] Maret W. Sandstead H. H. (2006). Zinc requirements and the risks and benefits of zinc supplementation. J. Trace Elem. Med. Biol. 20, 3–18. 10.1016/j.jtemb.2006.01.006, PMID: 16632171

[ref45] Mozafar A. (1989). Boron effect on mineral nutrients of maize. Agron. J. 81, 285–290. 10.2134/agronj1989.00021962008100020029x

[ref46] MSZ-08-0206/2 (1978). Evaluation of some chemical properties of the soil. Laboratory tests. (pH value, phenolphtalein alkalinity expressed in soda, total water soluble salt content, hydrolytic (y1 value) and exchangeable acidity (y2 value). edited by Hungarian Standard Association. Hungary.

[ref47] MSZ-08-0215 (1978). Determination of the cation adsorption capacity of the soil. Modified mechlich technique. Hungarian Standard Association.

[ref48] MSZ-08-0452 (1980). Use of high-capacity analyser systems for soils analyses. Quantitative determination of the organic carbon content of the soil on Contiflo analyzer system. Hungarian Standard Association.

[ref49] MSZ-20135 (1999). Determination of the soluble nutrient element content of the soil. Hungarian Standard Association.

[ref50] MSZ-21470-50 (2006). Environmental testing of soils. Determination of total and soluble toxic element, heavy metal and chromium (VI) content. Hungarian Standard Association.

[ref51] Nable R. O. Bañuelos G. S. Paull J. G. (1997). Boron toxicity. Plant Soil 193, 181–198. 10.1023/A:1004272227886

[ref52] Naghii M. R. Wall P. M. Samman S. (1996). The boron content of selected foods and the estimation of its daily intake among free-living subjects. J. Am. Coll. Nutr. 15, 614–619. 10.1080/07315724.1996.10718638 8951740

[ref53] Nielsen F. H. (1996). Evidence for the nutritional essentiality of boron. J. Trace Elem. Exp. Med. 9, 215–229. 10.1002/(SICI)1520-670X(1996)9:4<215::AID-JTRA7>3.0.CO;2-P

[ref54] Nielsen F. H. (1997). Boron in human and animal nutrition. Plant Soil 193, 199–208. 10.1023/A:1004276311956

[ref55] Padilla F. M. de Souza R. Peña-Fleitas M. T. Grasso R. Gallardo M. Thompson R. B. (2019). Influence of time of day on measurement with chlorophyll meters and canopy reflectance sensors of different crop N status. Precis. Agric. 20, 1087–1106. 10.1007/s11119-019-09641-1

[ref56] Pandey N. Archana. (2013). Antioxidant responses and water status in Brassica seedlings subjected to boron stress. Acta Physiol. Plant. 35, 697–706. 10.1007/s11738-012-1110-z

[ref57] Pang J. Zhou M. Mendham N. Shabala S. (2004). Growth and physiological responses of six barley genotypes to waterlogging and subsequent recovery. Aust. J. Agric. Res. 55, 895–906. 10.1071/AR03097

[ref58] Parks J. L. Edwards M. (2005). Boron in the environment. Crit. Rev. Environ. Sci. Technol. 35, 81–114. 10.1080/10643380590900200

[ref59] Parks R. Q. Lyon C. B. Hood S. L. (1944). Some effects of boron supply on the chemical composition of tomato leaflets. Plant Physiol. 19, 404–419. 10.1104/pp.19.3.404, PMID: 16653926 PMC438171

[ref60] Pereira G. L. Siqueira J. A. Batista-Silva W. Barcellos Cardoso F. Nunes-Nesi A. Araújo W. L. (2021). Boron: more than an essential element for land plants? Front. Plant Sci. 11:610307. 10.3389/fpls.2020.610307, PMID: 33519866 PMC7840898

[ref61] Rainey C. J. Nyquist L. A. Christensen R. E. Strong P. L. Culver B. D. Coughlin J. R. (1999). Daily boron intake from the American diet. J. Am. Diet. Assoc. 99, 335–340. 10.1016/S0002-8223(99)00085-1 10076586

[ref62] Ramesh K. V. Paul V. Pandey R. (2020). Dynamics of mineral nutrients in tomato (*Solanum lycopersicum* L.) fruits during ripening: part I—on the plant. Plant Physiol. Rep. 26, 23–37. 10.1007/s40502-020-00546-0

[ref81] R Core Team (2019). R: A Language and Environment for Statistical Computing. Vienna: R Foundation for Statistical Computing.

[ref63] Redondo-Nieto M. Rivilla R. El-Hamdaoui A. Bonilla I. Bolaños L. (2001). Research note: boron deficiency affects early infection events in the pea-rhizobium symbiotic interaction. Funct. Plant Biol. 28, 819–823. 10.1071/PP01020

[ref64] Rees R. Robinson B. H. Menon M. Lehmann E. Günthardt-Goerg M. S. Schulin R. (2011). Boron accumulation and toxicity in hybrid poplar (*Populus nigra* × euramericana). Environ. Sci. Technol. 45, 10538–10543. 10.1021/es201100b, PMID: 22050628

[ref65] Rhoades J. D. (1972). Quality of water for irrigation. Soil Sci. 113, 277–284. 10.1097/00010694-197204000-00007

[ref66] Roessner U. Patterson J. H. Forbes M. G. Fincher G. B. Langridge P. Bacic A. (2006). An investigation of boron toxicity in barley using metabolomics. Plant Physiol. 142, 1087–1101. 10.1104/pp.106.084053, PMID: 16998089 PMC1630719

[ref67] Sarkar S. Banerjee H. Ray K. Ghosh D. (2018). Boron fertilization effects in processing grade potato on an inceptisol of West Bengal, India. J. Plant Nutr. 41, 1456–1470. 10.1080/01904167.2018.1457685

[ref68] Sasmaz A. (2008). Translocation and accumulation of boron in roots and shoots of plants grown in soils of low boron concentration in Turkey’s Keban Pb-Zn mining area. Int. J. Phytorem. 10, 302–310. 10.1080/15226510802096119, PMID: 19260215

[ref69] Sebastian A. Frassetto L. A. Sellmeyer D. E. Morris R. C. Jr. (2006). The evolution-informed optimal dietary potassium intake of human beings greatly exceeds current and recommended intakes. Semin. Nephrol. 26, 447–453. 10.1016/j.semnephrol.2006.10.003, PMID: 17275582

[ref70] Singh B. P. Singh B. (1990). Response of French bean to phosphorus and boron in acid Alfisols in Meghalaya. J. Indian Soc. Soil Sci. 38, 769–771.

[ref71] Tariq M. Mott C. J. B. (2006). Effect of boron supply on the uptake of micronutrients by radish (*Raphanus sativus* L.). J. Agric. Biol. Sci. 1, 1–8.

[ref72] Tariq M. Mott C. J. B. (2007). The significance of boron in plant nutrition and environment-a review. J. Agron. 6, 1–10. 10.3923/ja.2007.1.10

[ref73] True R. H. Hogan J. M. Augustin J. Johnson S. J. Teitzel C. Toma R. B.. (1978). Mineral composition of freshly harvested potatoes. Am. Potato J. 55, 511–519. 10.1007/BF02852157

[ref74] Turan M. A. Taban N. Taban S. (2009). Effect of calcium on the alleviation of boron toxicity and localization of boron and calcium in cell wall of wheat. Not. Bot. Hort. Agrobot. Cluj-Napoca 37, 99–103. 10.15835/nbha3723241

[ref75] Türker O. C. Yakar A. Türe C. Saz Ç. (2019). Cost-effectiveness of boron (B) removal from irrigation water: an economic water treatment model (EWTM) for farmers to prevent boron toxicity. Environ. Sci. Pollut. Res. 26, 18777–18789. 10.1007/s11356-019-05268-x, PMID: 31062239

[ref76] Wang C. Ji J. Chen M. Zhong C. Yang Z. Browne P. (2017). Atmospheric contribution to boron enrichment in aboveground wheat tissues. Chemosphere 174, 655–663. 10.1016/j.chemosphere.2017.01.124, PMID: 28199942

[ref77] Wear J. I. Patterson R. M. (1962). Effect of soil pH and texture on the availability of water-soluble boron in the soil. Soil Sci. Soc. Am. J. 26, 344–346. 10.2136/sssaj1962.03615995002600040011x

[ref78] White P. J. Bradshaw J. E. Finlay M. Dale B. Gavin R. Hammond J. P.. (2009). Relationships between yield and mineral concentrations in potato tubers. HortScience 44, 6–11. 10.21273/HORTSCI.44.1.6

[ref79] Wilcox L. V. (1960). Boron Injury to Plants. Washington, DC: Agricultural Information Bulletin 211, USDA-ARS.

