# Supplementary material for: Effect of the Boron Concentration in Irrigation Water on the Elemental Composition of Edible Parts of Tomato, Green Bean, Potato, and Cabbage Grown on Soils With Different Textures
Source: Front Plant Sci. 2021 Jun 14;12:658892. doi: 10.3389/fpls.2021.658892 (PMC8236942; doi:10.3389/fpls.2021.658892)
Supplement: Supplementary file 1 [file Data_Sheet_1.docx]

Suppl. Table 1. Settings of the ICP-MS instrument.

| **Plasma power** | 1290 W |
| --- | --- |
| **Outer gas (Ar)** | 7.5 L/min |
| **Intermediate gas (Ar)** | 1.5 L/min |
| **Aerosol carrier gas (Ar)** | 1.0 L/min |
| **Reaction gas (He)** | 90 mL/min |
| **Reaction gas (H_2_)** | 110 mL/min |
| **Sample uptake** | 0.30 mL/min |
| **Nebulizer** | Meinhard |
| **Spray chamber** | double pass |
| **Sampler cone** | Ni. 1.1 mm orifice |
| **Skimmer cone** | Ni. 0.5 mm orifice |
| **Analytical isotopes** | ^11^B; ^26^Mg;^39^K; ^56^Fe; ^63^Cu;^66^Zn |
| **Internal standards** | ^45^Sc; ^89^Y;^115^In;^159^Tb |
| **Data acquisition** | peak jumping |
| **Dwell time** | 30 ms |
| **Replicates** | 5x20 |

Suppl. Table 2. Plant-available (ammonium acetate + EDTA-soluble) B content of soils after treatment with different rates of B irrigation (mg/kg) Data are the mean ±sd of the replicates. Different letters indicate significant differences between rows (p < 0.05).

| **Soil** | **Irraigation water** | **Bean** | **Cabbage** | **Tomato** | **Potato** |
| --- | --- | --- | --- | --- | --- |
| **Sand** | **Control** | 0.845 ±0.061 c | 0.508 ±0.330 bc | 0.790 ±0.004 b | 0.818 ±0.031 abc |
|  | **0.1 mg B/l** | 0.820 ±0.006 c | 0.777 ±0.057 c | 0.963 ±0.062 c | 0.859 ±0.011 abc |
|  | **0.5 mg B/l** | 1.035 ±0.087 a | 1.359 ±0.117 d | 1.498 ±0.055 d | 1.081 ±0.070 ab |
| **Silty sand** | **Control** | 0.464 ±0.001 b | 0.100 ±0.005 a | 0.091 ±0.024 a | 0.347 ±0.227 d |
|  | **0.1 mg B/l** | 0.500 ±0.009 b | 0.101 ±0.024 a | 0.093 ±0.025 a | 0.538 ±0.040 cd |
|  | **0.5 mg B/l** | 0.548 ±0.064 b | 0.152 ±0.027 a | 0.159 ±0.025 a | 0.704 ±0.051 bcd |
| **Silt** | **Control** | 0.949 ±0.071 ac | 0.150 ±0.003 a | 0.127 ±0.022 a | 0.944 ±0.124 ab |
|  | **0.1 mg B/l** | 1.034 ±0.094 a | 0.164 ±0.009 a | 0.158 ±0.008 a | 1.134 ±0.262 a |
|  | **0.5 mg B/l** | 1.072 ±0.087 a | 0.210 ±0.007 ab | 0.147 ±0.064 a | 1.195 ±0.106 a |

Suppl. Table 3. Weight of tomato and green bean plant parts dry weight and fruit fresh weight (g/plant). Data are the mean ±sd of the replicates. Different letters indicate significant differences between rows (p < 0.05).

| **Soil** | **Irraigation water** | **Tomato** | | | | **Green Bean** | | | |
| --- | --- | --- | --- | --- | --- | --- | --- | --- | --- |
|  |  | **Root** | **Shoot** | **Fruit (dry)** | **Fruit (fresh)** | **Root** | **Shoot** | **Fruit (dry)** | **Fruit (fresh)** |
| **Sand** | **Control** | 2.96 ±0.16 a | 23.7 ±3.6 a | 24.8 ±2.9 a | 372 ±58 a | 4.10 ±1.36 b | 16.0 ±4.1 a | 8.2 ±2.6 bc | 86.9 ±19.9 ab |
|  | **0.1 mg B/l** | 2.68 ±0.43 a | 24.7 ±5.0 a | 18.8 ±3.8 a | 269 ±38 a | 3.50 ±0.61 bc | 14.0 ±3.3 a | 9.2 ±0.9 abc | 99.4 ±9.2 a |
|  | **0.5 mg B/l** | 3.03 ±0.52 a | 26.2 ±5.4 a | 18.3 ±5.3 a | 318 ±97 a | 3.77 ±0.31 b | 10.3 ±2.0 a | 4.5 ±3.5 c | 47.9 ±38.7 b |
| **Silty sand** | **Control** | 2.52 ±0.21 a | 24.9 ±1.4 a | 19.1 ±10.1 a | 301 ±162 a | 1.64 ±0.14 a | 12.3 ±0.4 a | 13.6 ±0.5 a | 123.0 ±8.0 a |
|  | **0.1 mg B/l** | 4.00 ±0.98 a | 36.1 ±5.4 a | 20.2 ±3.8 a | 265 ±70 a | 1.58 ±0.15 a | 11.6 ±1.9 a | 14.3 ±0.6 a | 127.7 ±0.7 a |
|  | **0.5 mg B/l** | 2.68 ±0.29 a | 26.2 ±2.2 a | 23.3 ±4.0 a | 352 ±40 a | 1.67 ±0.10 a | 11.9 ±2.2 a | 13.1 ±0.4 ab | 103.3 ±9.0 a |
| **Silt** | **Control** | 4.16 ±0.69 a | 31.7 ±6.1 a | 20.6 ±5.9 a | 304 ±120 a | 2.01 ±0.18 ac | 13.2 ±0.6 a | 11.3 ±1.6 ab | 106.1 ±11.0 a |
|  | **0.1 mg B/l** | 3.25 ±0.62 a | 23.3 ±12.6 a | 20.0 ±2.8 a | 329 ±75 a | 1.56 ±0.29 a | 10.2 ±2.1 a | 10.8 ±2.4 ab | 110.3 ±16.9 a |
|  | **0.5 mg B/l** | 3.82 ±0.91 a | 19.7 ±7.7 a | 14.4 ±2.8 a | 242 ±28 a | 2.60 ±0.17 abc | 11.6 ±2.1 a | 10.7 ±0.5 ab | 99.3 ±7.0 a |

Suppl. Table 3 cont. Weight of potato and cabbage plant parts dry weight and potato tuber and cabbage shoot fresh weight (g/plant). Data are the mean ±sd of the replicates. Different letters indicate significant differences between rows (p < 0.05).

| **Soil** | **Irraigation water** | **Potato** | | | | **Cabbage** | | |
| --- | --- | --- | --- | --- | --- | --- | --- | --- |
|  |  | **Root** | **Shoot** | **Tuber (dry)** | **Tuber (fresh)** | **Root** | **Shoot (dry)** | **Shoot (fresh)** |
| **Sand** | **Control** | 2.42 ±0.23 ab | 9.72 ±0.86 ab | 38.1 ±2.0 a | 192 ±7 a | 1.95 ±0.9 a | 42.9 ±18.4 ab | 510 ±226 a |
|  | **0.1 mg B/l** | 2.62 ±0.09 ab | 9.97 ±0.48 ab | 37.7 ±2.0 a | 188 ±10 a | 2.50 ±0.5 a | 66.6 ±9.9 b | 580 ±29 a |
|  | **0.5 mg B/l** | 2.47 ±1.29 ab | 8.11 ±2.70 ab | 24.3 ±14.1 a | 130 ±72 a | 4.34 ±0.3 b | 54.3 ±4.2 ab | 461 ±55 a |
| **Silty sand** | **Control** | 2.29 ±0.23 ab | 8.98 ±1.11 ab | 36.7 ±2.7 a | 191 ±16 a | 1.40 ±0.4 a | 38.9 ±9.7 a | 476 ±127 a |
|  | **0.1 mg B/l** | 2.55 ±0.83 ab | 8.83 ±1.07 ab | 37.1 ±2.8 a | 189 ±8 a | 1.69 ±0.5 a | 55.9 ±6.6 ab | 602 ±8 a |
|  | **0.5 mg B/l** | 1.73 ±0.36 a | 7.23 ±0.85 a | 28.1 ±7.7 a | 167 ±33 a | 2.06 ±0.5 a | 48.8 ±6.9 ab | 510 ±132 a |
| **Silt** | **Control** | 3.31 ±1.28 ab | 11.00 ±2.20 ab | 36.8 ±0.3 a | 191 ±8 a | 2.17 ±0.3 a | 56.0 ±2.2 ab | 559 ±44 a |
|  | **0.1 mg B/l** | 3.87 ±0.56 b | 11.19 ±0.09 b | 38.3 ±2.4 a | 184 ±12 a | 2.97 ±1.0 ab | 57.3 ±4.3 ab | 482 ±44 a |
|  | **0.5 mg B/l** | 3.40 ±0.18 ab | 9.38 ±0.27 ab | 30.4 ±5.3 a | 181 ±5 a | 2.80 ±0.4 ab | 57.9 ±5.1 ab | 501 ±37 a |
